# Supplementary figures and images for: miR-1293 acts as a tumor promotor in lung adenocarcinoma via targeting phosphoglucomutase 5
Source: PeerJ. 2021 Sep 16;9:e12140. doi: 10.7717/peerj.12140 (PMC8450003; doi:10.7717/peerj.12140)

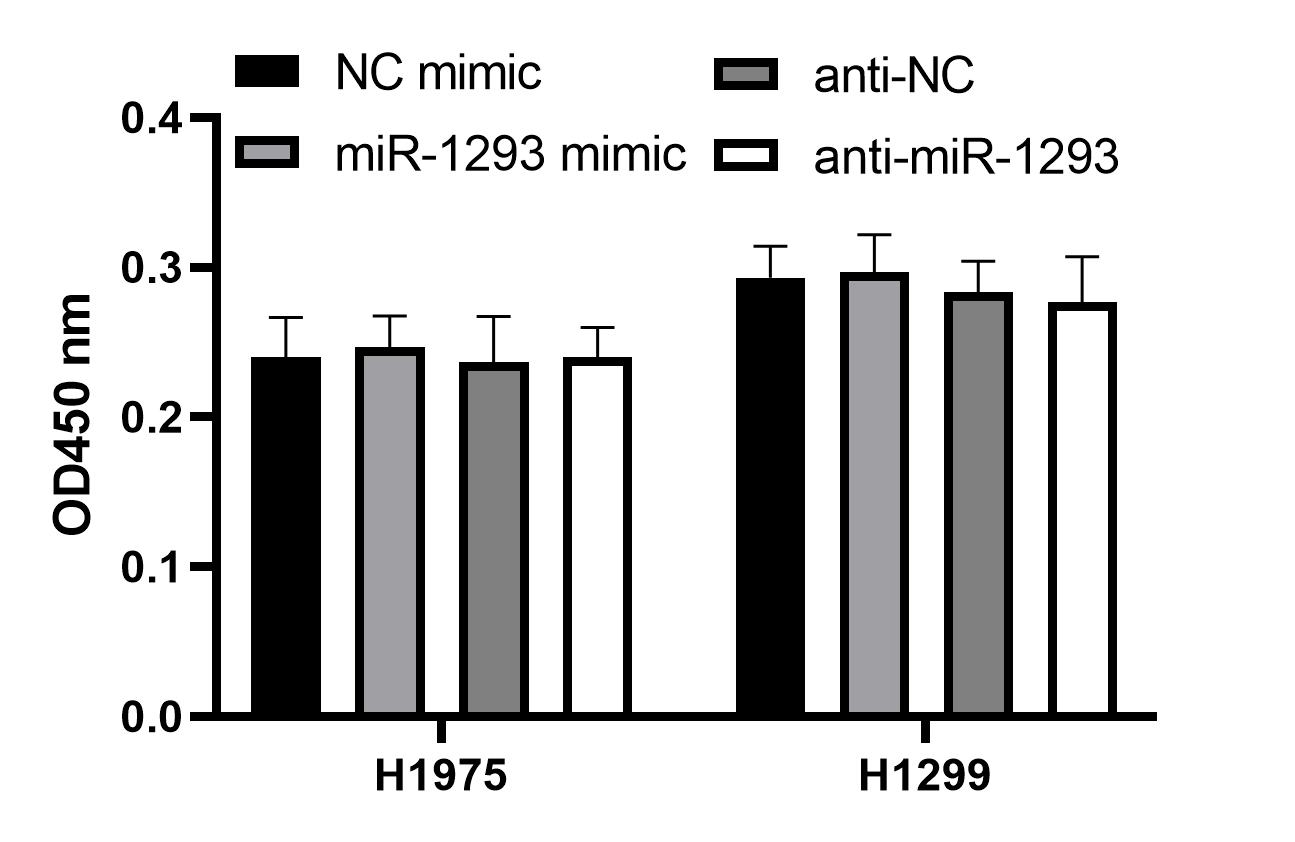

Supplement: Supplemental Information 4 — H1975 and H1299 cells transfected with miR-1293 mimic or antagomir (anti-miR-1293) were seeded into 96-well plates and cultured in serum-free RPMI 1640 medium for 48 h, cell viability was analyzed by CCK-8 assay. [file peerj-09-12140-s004.jpg]
